# A Review on the Carcinogenic Roles of DSCAM-AS1

**DOI:** 10.3389/fcell.2021.758513

**Published:** 2021-10-11

**Authors:** Soudeh Ghafouri-Fard, Tayyebeh Khoshbakht, Mohammad Taheri, Kaveh Ebrahimzadeh

**Affiliations:** ^1^Department of Medical Genetics, School of Medicine, Shahid Beheshti University of Medical Sciences, Tehran, Iran; ^2^Men’s Health and Reproductive Health Research Center, Shahid Beheshti University of Medical Sciences, Tehran, Iran; ^3^Urology and Nephrology Research Center, Shahid Beheshti University of Medical Sciences, Tehran, Iran; ^4^Skull Base Research Center, Loghman Hakim Hospital, Shahid Beheshti University of Medical Sciences, Tehran, Iran

**Keywords:** DSCAM-AS1, M41, lncRNA, cancer, biomarker

## Abstract

Long non-coding RNAs (lncRNAs) are a group of transcripts with fundamental roles in the carcinogenesis. DSCAM Antisense RNA 1 (DSCAM−AS1) is an example of this group of transcripts which has been firstly identified in an attempt to find differentially expressed transcripts between breast tumor cells and benign breast samples. The pathogenic roles of DSCAM-AS1 have been vastly assessed in breast cancer, yet its roles are not restricted to this type of cancer. Independent studies in non-small cell lung cancer, colorectal cancer, osteosarcoma, hepatocellular carcinoma, melanoma and cervical cancer have validated participation of DSCAM-AS1 in the carcinogenic processes. miR-577, miR-122-5p, miR-204-5p, miR-136, miR−137, miR−382, miR−183, miR−99, miR-3173-5p, miR-874-3p, miR-874-3p, miR-150-5p, miR-2467-3p, miR-216b, miR-384, miR-186-5p, miR-338-3p, miR-877-5p and miR-101 are among miRNAs which interact with DSCAM-AS1. Moreover, this lncRNA has interactions with Wnt/β-catenin pathway. The current study aims at summarization of the results of studies which focused on the assessment of oncogenic role of DSCAM-AS1.

## Introduction

Long non-coding RNAs (lncRNAs) have recently considerable attention among molecular oncologists because of their vast and pervasive impacts in the process of carcinogenesis (Carlevaro-Fita et al., 2020). Up to now, tens of thousands of lncRNAs have been identified (Derrien et al., 2012). They have sizes > 200 nt, yet they do not principally make functional proteins. Moreover, they are evolutionary conserved and are strictly regulated (Ulitsky and Bartel, 2013). Through establishing complexes with proteins and RNAs, they regulate expression of genes not only within the nucleus but also outside the nuclear compartment (Guttman and Rinn, 2012).

DSCAM Antisense RNA 1 (DSCAM-AS1) is an example of this group of transcripts which has been firstly described by Liu et al. (2002) in an attempt to find differentially expressed transcripts between benign and malignant breast tumor cells. Authors have described this transcript as an estrogen-responsive expressed sequence tag being transcribed from an intronic region on chromosome 21q22.3 (Liu et al., 2002). Up to now, four splice variants have been reported for this lncRNA with sizes of 1,640, 1,228, 1,185, and 1,153, respectively^1^.

Following the research conducted by Liu et al. (2002), Miano et al. (2016) have reported DSCAM-AS1 as the most abundant Apo−Estrogen Receptor α−regulated lncRNA in MCF−7 breast cancer cells. Notably, this lncRNA has been recognized as the main distinguishing feature of the luminal subtype of breast cancer (Miano et al., 2016). A subsequent study has demonstrated interaction between DSCAM−AS1 and hnRNPL in the context of breast cancer. Such interaction has been found to facilitate progression of breast cancer and induce resistance to tamoxifen (Niknafs et al., 2016). After these pioneering studies in breast cancer, several studies have appraised the expression levels of DSCAM-AS1 in different types of malignancies. Since this lncRNA has been dysregulated in several types of cancers, it might be used as a diagnostic marker or therapeutic target for a wide range of neoplastic conditions. Thus, it is necessary to unravel the mechanisms underlying DSCAM-AS1 dysregulation and the functional consequences of this dysregulation. The current study aims at summarization of the results of these studies.

## Cell Line Experiments

A set of experiments in different cancer cell lines has shown that DSCAM-AS1 expression is regulated by two super-enhancers induced by FOXA1. DSCAM-AS1 has been shown to influence expression of the principal transcriptional factor FOXA1. In MCF-7 breast cancer cells, DSCAM-AS1 could affect expression of estrogen receptor α (ERα). Functionally, DSCAM-AS1 interplays with YBX1 and affects recruitment of YBX1 to FOXA1 and ERα promoters (Zhang et al., 2020b).

### DSCAM-AS1 Expression in Lung Cancer Cell Lines

DSCAM-AS1 has been found to be up-regulated in lung cancer cells parallel with up-regulation of HMGB1 and down-regulation of miR-577. DSCAM-AS1 has an established role in enhancement of proliferation, migratory aptitude and invasive properties of lung cancer cells. Functionally, DSCAM-AS1 regulates expression of HMGB1 through binding with miR-577 and sequestering it. Through miR-577/HMGB1 axis, DSCAM-AS1 could also regulate activity of Wnt/β-catenin pathway (Qiu et al., 2020). Another way of participation of DSCAM-AS1 in the pathogenesis of lung cancer is mediated through up-regulation of BCL11A (Liao and Xie, 2019), a proto-oncogene which is activated in lung cancer through different mechanisms such as gene amplification and over-expression of miR-30a (Jiang et al., 2013). Thus, DSCAM-AS1 establishes a less-appreciated route of proto-oncogene over-expression in lung cancer. Besides, DSCAM-AS1 can decrease bioavailability of miR-122-5p, thus releasing FSTL3 from its inhibitory effects. Since FSTL3 is an oncogene in lung cancer, DSCAM-AS1-mediated up-regulation of this oncogene can promote carcinogenesis process in this type of tissue (Gao et al., 2020).

### DSCAM-AS1 Expression in Breast Cancer Cell Lines

In breast cancer cells, up-regulation of DSCAM-AS1 has been associated with reduction of miR-204-5p. The direct interplay between DSCAM-AS1 and miR-204-5p has also been verified. Pro-proliferation and pro-invasion effects of DSCAM-AS1 in breast cancer have been found to be mediated through inhibition of miR-204-5p and subsequent up-regulation of RRM2 (Liang et al., 2019). DSCAM-AS1 silencing in breast cancer cells has led to alteration of more than 900 genes which have been mostly related with regulation of cell cycle and immune responses. Most notably, more than 2,000 splicing events have been shown to be regulated by DSCAM-AS1. Among these events have been alternative polyadenylation events, shortened 3′UTR and exon skipping events. The splicing factor hnRNPL has been demonstrated to interact with DSCAM-AS1 and mediate exon skipping and 3′UTR shortening events (Elhasnaoui et al., 2020). DSCAM-AS1 has also been reported to increase Tamoxifen resistance in breast cancer cells *via* sponging miR-137, then increasing expression of EPS8. miR-137 can prompt cell cycle arrest at the G0/G1 phase, so its suppression by DSCAM-AS1 leads to enhancement of cell reproduction and inhibition of cell apoptosis in tamoxifen resistant breast cancer cells (Ma et al., 2019).

### DSCAM-AS1 Expression in Colon Cancer Cell Lines

In colon cancer, DSCAM-AS1 can down-regulate expression of miR-216b to enhance the migratory potential and invasion of cancer cells (Liu et al., 2019). Moreover, in this type of cancer, DSCAM-AS1 serves as a molecular sponge for miR-384 to enhance expression of AKT3 (Li et al., 2020). The sponging effect of DSCAM-AS1 on miR-204 and subsequent activation of SOX4 is another rout of participation of DSCAM-AS1 in the pathoetiology of colon cancer (Lu et al., 2020).

Another study in colorectal cancer cells has shown the sponging effect of DSCAM-AS1 on miR-137 (Xu et al., 2020). This miRNA has been found to suppress expression of Notch-1, a protein with essential roles in cell proliferation and epithelial-mesenchymal transition (EMT) (Chu et al., 2019). Suppression of DSCAM-AS1 expression in colorectal cancer cells has resulted in down-regulation of Notch-1 (Xu et al., 2020).

### DSCAM-AS1 Expression in Osteosarcoma Cell Lines

The oncogenic roles of DSCAM-AS1 in osteosarcoma have been validated through different investigations. DSCAM-AS1 silencing has considerably inhibited viability and invasive properties of osteosarcoma cells, whereas DSCAM-AS1 up-regulation has exerted the opposite effects. DSCAM-AS1 has also been found to inhibit miR-101 expression through directly interacting with its 3′UTR (Yu et al., 2020). Another study has confirmed interaction between DSCAM-AS1 and miR-101-3p and the resultant up-regulation of USP47 in osteosarcoma (Zhang et al., 2020a). Finally, DSCAM-AS1 can promote proliferation and migration of malignant cells *via* modulation of miR-186-5p/GPRC5A cascade (Ning and Bai, 2021).

### DSCAM-AS1 Expression in Other Cancer Cell Lines

**FIGURE 1:**
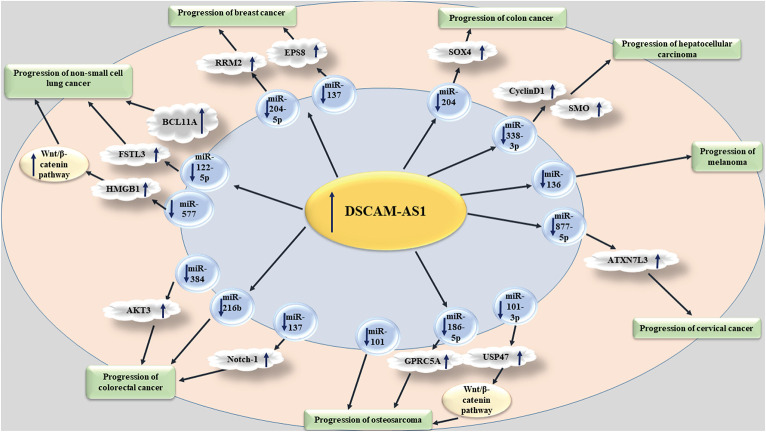
The effects of DSCAM-AS1 in different types of cancers.

DSCAM-AS1 has sponging effects on a variety of other miRNAs such as miR-338-3p, miR-136 and miR-877-5p. In hepatocellular carcinoma cells, DSCAM-AS1 can enhance proliferation, migration and invasion. These effects of DSCAM-AS1 have been found to be mediated through sponging miR-338-3p, a miRNA that can regulate expressions of both CyclinD1 and SMO (Ji et al., 2019). DSCAM-AS1 has also a prominent role in the pathogenesis of melanoma through interacting with miR-136 (Huang et al., 2019). In cervical cancer cells, DSCAM-AS1 interacts with miR-877-5p to increase expression of its target gene ATXN7L3 (Liang et al., 2020). DSCAM-AS1 has also been found to be up-regulated in gastric cancer cell lines. DSCAM-AS1 knock-down has reduced proliferation and migration of these cells. DSCAM-AS1 sequesters miR-204 in these cells, thus increasing expression of its target i.e., TPT1 (Wang et al., 2021) (Figure 1).

Table 1 summarizes the results of *in vitro* assessments of DSCAM-AS1 roles in cancer.

**TABLE 1:** Outlines of researches which judged expression of DSCAM-AS1 in cell lines (Δ: knock-down or deletion).

**Tumor type**	**Targets/Regulators and signaling pathways**	**Cell line**	**Function**	**References**
Non-small cell lung cancer	miR-577, HMGB1, Wnt/β-catenin pathway	A549, H1299, H460, BEAS-2B	Δ DSCAM-AS1: ↓ proliferation, ↓ migration, ↓ invasion, ↑ apoptosis	Qiu et al., 2020
	BCL11A	SPCA1, A549, PC-9, H1975, 16HBE	↑ DSCAM-AS1: ↑ migration,↑invasion	Liao and Xie, 2019
	miR-122-5p, FSTL3	16HBE, A549, NCI-H460, H1299, L9981, NCI-H292	↑DSCAM-AS1:↑ proliferation,↑ migration	Gao et al., 2020
Breast cancer	miR-204-5p, RRM2	HCC1937	↑DSCAM-AS1:↑proliferation,↑ migration,↑invasion,↑metastasis, ↓ apoptosis	Liang et al., 2019
	hnRNPL	MCF-7, SK-BR-3	Δ DSCAM-AS1: ↓ proliferation, significant changes in isoform switching events	Elhasnaoui et al., 2020
	miR−137, EPS8	MCF7, T47D, SK−BR−3, MDA−MB−31	Δ DSCAM-AS1: ↓ proliferation, ↓ tamoxifen resistance,↑G0/G1 phase arrest	Ma et al., 2019
	miR−382, miR−183, miR−99	MCF−7, T−47D	Δ DSCAM-AS1: ↓ proliferation, ↓ colony formation,↑G1/S phase arrest	Sun et al., 2018
	DCTPP1, QPRT, miR-3173-5p, miR-874-3p, miR-874-3p, miR-150-5p and miR-2467-3p	MCF-7, T47D	Δ DSCAM-AS1: ↓ proliferation, ↓ migration, ↓ invasion,↑ apoptosis	Yue et al., 2020
	_	MCF10A, MDA-MB-231	_	Yin et al., 2021
Colorectal cancer	miR-216b	WiDr, HT-29	↑ DSCAM-AS1:↑ migration,↑ invasion, did not significantly affect proliferation	Liu et al., 2019
	miR-384, AKT3	NCM460, LOVO, PKO, SW480, HT29	Δ DSCAM-AS1: ↓ proliferation, ↓ migration, ↓ invasion	Li et al., 2020
	miR-137, Notch-1	HT29, LOVO, SW480, PKO, NCM460	Δ DSCAM-AS1: ↓ proliferation, ↓ migration	Xu et al., 2020
	miR-204, SOX4	HT29, HCT8, SW480, LOVO, NCM460	Δ DSCAM-AS1: ↓ proliferation, ↓ migration	Lu et al., 2020
Osteosarcoma	miR-101	hFOB, U2OS, SAOS2, HOS	Δ DSCAM-AS1: ↓ viability, ↓ invasion ↑ DSCAM-AS1:↑ viability,↑ invasion	Yu et al., 2020
	miR-101-3p, USP47, Wnt-β-catenin signaling pathway	_	Δ DSCAM-AS1: ↓ proliferation, ↓ migration, ↓ invasion,↑ apoptosis	Zhang et al., 2020a
	miR-186-5p, GPRC5A	_	Δ DSCAM-AS1: ↓ proliferation, ↓ migration, ↓ EMT process	Ning and Bai, 2021
Hepatocellular carcinoma	miR-338-3p, CyclinD1, SMO	LO2, HepG2, Hep3B, Huh7, SMMC7721	Δ DSCAM-AS1: ↓ proliferation, ↓ migration, ↓ invasion	Ji et al., 2019
Melanoma	miR-136	1205Lu, CHL-1, A-375, UACC903, SK-MEL-2, HEMa-LP	Δ DSCAM-AS1: ↓ proliferation, ↓ migration, ↓ invasion,↑ apoptosis	Huang et al., 2019
Cervical cancer	miR-877-5p, ATXN7L3	H8, SiHa, HeLa, C-33A, CaSki	Δ DSCAM-AS1: ↓ proliferation, ↓ migration, ↓ invasion	Liang et al., 2020

## Animal Studies

The functional role of DSCAM-AS1 in the carcinogenesis has been verified through knock-down studies in xenograft models of lung, breast and colorectal cancers (Table 2). All studies have confirmed that DSCAM-AS1 knock-down in cancer cell lines diminishes their ability to make tumors, thus decreasing tumor volume and weight. Two additional studies in lung cancer (Gao et al., 2020) and HCC (Ji et al., 2019) have shown that knock-down of DSCAM-AS1 downstream target FSTL3 similarly decreases tumor volume. Moreover, in xenograft tumors generated from DSCAM-AS1-suppressed colorectal cancer cells, AKT3 expression has been shown to be decreased, while miR-384 level has been increased, demonstrating the role of DSCAM-AS1 in enhancement of AKT3 levels through modulation of expression of miR-384 (Li et al., 2020).

**TABLE 2:** Results of studies which evaluated function of DSCAM-AS1 in animal models (Δ: knock-down or deletion).

**Tumor type**	**Animal models**	**Results**	**References**
Non-small cell lung cancer	male BALB/c nude mice	Δ DSCAM-AS1: ↓ tumor volume, ↓ tumor weight	Qiu et al., 2020
	female BALB/c nude mice	Δ FSTL3: ↓ tumor weight, ↓ metastasis	Gao et al., 2020
Breast cancer	female nude mice	↑ DSCAM-AS1:↑ tumor volume,↑ tumor weight Δ DSCAM-AS1: ↓ tumor volume, ↓ tumor weight	Liang et al., 2019
	female BALB/c nude mice		Ma et al., 2019
	male BALB/c nude mice		Yue et al., 2020
Colorectal cancer	male athymic nude mice	Δ DSCAM-AS1: ↓ tumor volume, ↓ tumor weight	Li et al., 2020
Hepatocellular carcinoma	male BALB/c nude mice	Δ FSTL3: ↓ tumor weight, ↓ tumor size, ↓ tumor growth	Ji et al., 2019

Subcutaneous injection of DSCAM-AS1-silenced H460 cells into nude mice has resulted in attenuation of tumor growth in xenograft models as being evident by significant decrease in tumor bulk and weight. Moreover, these tumors exhibited lower levels of HMGB1, while higher levels of miR-577 expression compared with controls (Qiu et al., 2020).

In xenograft model of breast cancer, DSCAM-AS1 silencing could decrease the tumorigenic potential of cancer cells and increase miR-204-5p levels (Liang et al., 2019).

## Clinical Investigations

Studies that assessed expression of DSCAM-AS1 in neoplastic tissues have consistently reported up-regulation of this lncRNA in malignant tissues compared with their normal counterparts (Table 3). For instance, DSCAM-AS1 has been found to be over-expressed in high grade Luminal A, B, and HER2 + breast cancer samples. Remarkably, over-expression of DSCAM-AS1 in these samples has been correlated with tumor relapse (Elhasnaoui et al., 2020). Moreover, expression of DSCAM1 has been higher in tamoxifen resistant breast cancer samples compared with non-resistant ones (Ma et al., 2019). A retrospective assessment of clinical data of patients with breast cancer has shown association between up-regulation of DSCAM-AS1 and poor prognosis in patients with luminal breast cancer received endocrine therapy. Thus, DSCAM-AS1 has been suggested as a possible target for enhancement of survival of this kind of breast cancer (Sun et al., 2018). In melanoma, up-regulation of DSCAM-AS1 has been associated with ulceration and advanced clinical stage, resulting in poor patients’ survival. The latter has been verified through univariate and multivariate analyses (Huang et al., 2019).

**TABLE 3:** Results of papers that reported dysregulation of DSCAM-AS1 in clinical specimens (ANCTs, adjacent non-cancerous tissues; OS, Overall survival; DFS, Disease-free survival; TNM, tumor−node−metastasis; ER, Estrogen Receptor; TR, Tamoxifen−resistant; WT, wild type; TNBC, triple negative breast cancer; RFS, Relapse Free Survival).

**Tumor type**	**Samples**	**Expression (Tumor vs. Normal)**	**Kaplan–Meier analysis (impact of DSCAM-AS1 up-regulation)**	**Univariate/Multivariate cox regression**	**Association of DSCAM-AS1 expression with Clinicopathologic characteristics**	**References**
Non-small cell lung cancer (NSCLC)	32 NSCLC tissues and ANCTs	High	_	_	_	Qiu et al., 2020
	56 tumor tissue samples	High	Worse OS	_	_	Liao and Xie, 2019
Breast cancer (BC)	40 BC tissues and ANCTs	High	_	_	_	Liang et al., 2019
	TCGA analysis: 30 microarray datasets	_	In 3 of nine datasets: a higher relapse rate, in the 6 remaining datasets: a non-significant lower RFS rate	_	ER + tumors, high grade Luminal A, B and HER2 + BC	Elhasnaoui et al., 2020
	42 BC samples	_	_	_	ER + tumors	
	51 BC samples	_	Significant difference in relapse rate	_	_	
	GEO analysis: (GSE5840)	Higher in TR BC cells than WT BC cells	_	_	_	Ma et al., 2019
	30 BC tissues	Higher in TR BC tissues than WT BC tissues	_	_	_	
	20 pairs of BC tissues and ANCTs	High	_	_	_	Moradi et al., 2020
	40 BC patients and 40 healthy controls	High	_	_	_	
	50 pairs of BC tissues and ANCTs	High	_	_	TNM stages and HER-2 positive status	Tahmouresi et al., 2020
	399 luminal BC patients	High	Shorter DFS	DSCAM−AS1 could be an independent prognostic factor.	_	Sun et al., 2018
	309 patients in endocrine therapy group and 90 patients in no endocrine therapy group	Weakly expressed in 125 patients and highly expressed in 184 patients (patients from endocrine therapy group)	Patients received endocrine therapy: worse DFS patients from no endocrine therapy group: no effect on DFS	DSCAM−AS1, grade, and positive lymph node number were found to be independent prognostic factors.	_	
	21 pairs of luminal BC tissues and ANCTs	High	_	_	Luminal and Her−2 overexpression BC tissues, but not in TNBC	
	50 pairs of BC tissues and ANCTs	High	_	_	Lymph node metastasis	Tarighi et al., 2021
	TCGA analysis: 1098 BC patients and 113 healthy controls	High	_	_	_	Yin et al., 2021
Colorectal cancer (CRC)	70 pairs of primary tumor tissues and ANCTs	High	Worse OS	_	Tumor metastasis	Liu et al., 2019
	56 CRC tissues and ANCTs	High	Shorter OS	_	Advanced clinical stage and lymph node metastasis	Li et al., 2020
	51 CRC tissues and ANCTs	High	Poorer OS	_	Metastasis status and advanced stage	Xu et al., 2020
	37 pairs of colon cancer tissues and ANCTs	High	Poorer OS	_	Clinical tumor stage and lymph node metastasis	Lu et al., 2020
Osteosarcoma	32 osteosarcoma tissues and ANCTs	High	Worse prognosis	_	TNM stage, lymph node metastases, and distant metastasis	Yu et al., 2020
Hepatocellular carcinoma (HCC)	48 pairs of HCC tissues and ANCTs	High	Worse OS	_	Vascular invasion and TNM stage	Ji et al., 2019
	48 HCC patients and 30 heath donors					
Melanoma	104 pairs of melanoma tissues and ANCTs	High	Poorer OS	High level of DSCAM-AS1 was independent prognostic factor of OS.	Ulceration and stage	Huang et al., 2019

Expression of DSCAM-AS1 has been reported to be up-regulated in lung cancer tissues compared with normal samples. Besides, up-regulation of this lncRNA has been correlated with up-regulation of HMGB1 in these tissues (Qiu et al., 2020). Another study in lung cancer has verified up-regulation of DSCAM-As1 in tumor samples and assessed the overall survival of these patients following surgery through Kaplan–Meier survival analysis showing correlation between DSCAM-AS1 up-regulation and poor overall survival of patients (Liao and Xie, 2019).

A single study in bladder cancer has reported similar levels of DSCAM-AS1 between tumoral and adjacent non-tumoral tissues (Abdolmaleki et al., 2020). Other studies have in different types of cancer validated correlation between DSCAM-AS1 over-expression and low survival rate in terms of overall, disease-free or relapse free survival times.

## DSCAM-AS1 and Drug Resistance

DSCAM-AS1 levels can affect response of patients to anti-cancer drugs. For instance, DSCAM-AS1 up-regulation can increase Tamoxifen resistance in breast cancer through sequestering miR-137, then increasing expression of EPS8 (Ma et al., 2019). DSCAM-AS1 has also been shown to increase expressions of DCTPP1 and QPRT, two proteins whose effects on DNA function are possibly associated with resistance to chemo/radiotherapy (Yue et al., 2020).

## Discussion

DSCAM-AS1 is an oncogenic lncRNA in various tissues. This lncRNA play a part in essential biological processes, such as DNA replication, cell cycle transition particularly at G1/S phase, sister chromatid unity at the onset of chromosome segregation, recruitment of proteins on the chromosomes and DNA recombination (Sun et al., 2018). Consistent with these diverse roles, up-regulation of DSCAM-AS1 has been associated with carcinogenic events. Its oncogenic effects are mediated through interaction with proteins and transcripts. Several miRNAs including miR-577, miR-122-5p, miR-204-5p, miR-136, miR−137, miR−382, miR−183, miR−99, miR-3173-5p, miR-874-3p, miR-874-3p, miR-150-5p, miR-2467-3p, miR-216b, miR-384, miR-186-5p, miR-338-3p, miR-877-5p and miR-101 have been found to be regulated by DSCAM-AS1. The interaction between DSCAM-AS1 and miR-137, miR-204 and miR-101 has been validated in different studies. Consistently, DSCAM-AS1 can decrease expression of several tumor suppressor miRNAs, thus releasing the oncogenic targets of these miRNAs from their inhibitory effects. Cumulatively, DSCAM-AS1 up-regulates several oncogenes through this mechanism.

miR-577/HMGB1, miR-122-5p/FSTL3, miR-204-5p/RRM2, miR-137/Notch1, miR-186-5p/GPRC5A, miR-877-5p/ATXN7L3, miR-384/AKT3 and miR-204/SOX4 are among molecular cascades being regulated by DSCAM-AS1. Based on these findings, Notch and AKT pathways are possibly regulated by DSCAM-AS1. In addition, Wnt/β-catenin is another cancer-related pathway which has been found to be functionally related with DSCAM-AS1.

In addition to serving as molecular sponge for miRNAs, DSCAM-AS1 can regulate carcinogenesis through modulation of alternative splicing and isoform regulation. Alternative polyadenylation events have been found to be correlated with development and progression of cancers (Zhang et al., 2020c). Moreover, 3′UTR shortening as another event associated with DSCAM-AS1 can repress expression of tumor-suppressor genes through disturbing competing endogenous RNA interaction (Park et al., 2018). Finally, a number of exon skipping events have been associated with cancers (Kim et al., 2020). Thus, DSCAM-AS1 represents an important therapeutic target in cancers being capable of affecting several cancer-related mechanisms.

The importance of DSCAM-AS1 up-regulation in deterioration of patients’ outcome has been validated in independent studies in breast, lung, colorectal, skin, bone and liver cancers potentiating this lncRNA as a prognostic marker. Further assessment of its expression in the circulation of patients with different cancer types is necessary to propose it as a non-invasive marker in this regard.

## Author Contributions

SG-F wrote and revised the draft. MT designed and supervised the study. KE and TK collected the data and designed the figures and tables. All authors read and approved the submitted version.

## Conflict of Interest

The authors declare that the research was conducted in the absence of any commercial or financial relationships that could be construed as a potential conflict of interest.

## Publisher’s Note

All claims expressed in this article are solely those of the authors and do not necessarily represent those of their affiliated organizations, or those of the publisher, the editors and the reviewers. Any product that may be evaluated in this article, or claim that may be made by its manufacturer, is not guaranteed or endorsed by the publisher.

## Footnotes

1
http://asia.ensembl.org/

## References

[B1] Abdolmaleki F. Ghafoui-Fard S. Taheri M. Mordadi A. Afsharpad M. Varmazyar S. (2020). Expression analysis of a panel of long non-coding RNAs (lncRNAs) revealed their potential as diagnostic biomarkers in bladder cancer. *Genomics* 112 677–682. 10.1016/j.ygeno.2019.04.020 31054930

[B2] Carlevaro-Fita J. Lanzós A. Feuerbach L. Hong C. Mas-Ponte D. Pedersen J. S. (2020). Cancer LncRNA Census reveals evidence for deep functional conservation of long noncoding RNAs in tumorigenesis. *Commun. Biol.* 3:36. 10.1038/s42003-019-0741-7 32024996 PMC7002399

[B3] Chu J. Y. Chau M. K. Chan C. C. Tai A. C. Cheung K. F. Chan T. M. (2019). miR-200c prevents TGF-β1-induced epithelial-to-mesenchymal transition and fibrogenesis in mesothelial cells by targeting ZEB2 and Notch1. *Mol. Ther. Nucleic Acids* 17 78–91. 10.1016/j.omtn.2019.05.008 31226520 PMC6586597

[B4] Derrien T. Johnson R. Bussotti G. Tanzer A. Djebali S. Tilgner H. (2012). The GENCODE v7 catalog of human long noncoding RNAs: analysis of their gene structure, evolution, and expression. *Genome Res.* 22 1775–1789. 10.1101/gr.132159.111 22955988 PMC3431493

[B5] Elhasnaoui J. Miano V. Ferrero G. Doria E. Leon A. E. Fabricio A. S. (2020). DSCAM-AS1-driven proliferation of breast cancer cells involves regulation of alternative exon splicing and 3′-end usage. *Cancers* 12:1453. 10.3390/cancers12061453 32503257 PMC7352480

[B6] Gao L. Chen X. Wang Y. Zhang J. (2020). Up-regulation of FSTL3, regulated by lncRNA DSCAM-AS1/miR-122-5p Axis, promotes proliferation and migration of non-small cell lung Cancer cells. *OncoTargets Ther.* 13:2725. 10.2147/OTT.S236359 32280246 PMC7131999

[B7] Guttman M. Rinn J. L. (2012). Modular regulatory principles of large non-coding RNAs. *Nature* 482 339–346. 10.1038/nature10887 22337053 PMC4197003

[B8] Huang Y. Xu Q. Wang X. (2019). Long noncoding RNA DSCAM-AS1 is associated with poor clinical prognosis and contributes to melanoma development by sponging miR-136. *Eur. Rev. Med. Pharmacol. Sci.* 23 2888–2897. 31002140 10.26355/eurrev_201904_17567

[B9] Ji D. Hu G. Zhang X. Yu T. Yang J. (2019). Long non-coding RNA DSCAM-AS1 accelerates the progression of hepatocellular carcinoma via sponging miR-338-3p. *Am. J. Transl. Res.* 11:4290. 31396335 PMC6684899

[B10] Jiang B.-Y. Zhang X.-C. Su J. Meng W. Yang X.-N. Yang J.-J. (2013). BCL11A overexpression predicts survival and relapse in non-small cell lung cancer and is modulated by microRNA-30a and gene amplification. *Mol. Cancer* 12:61. 10.1186/1476-4598-12-61 23758992 PMC3695801

[B11] Kim P. Yang M. Yiya K. Zhao W. Zhou X. (2020). ExonSkipDB: functional annotation of exon skipping event in human. *Nucleic Acids Res.* 48 D896–D907. 10.1093/nar/gkz917 31642488 PMC7145592

[B12] Li B. Sun H. Zhang J. (2020). LncRNA DSCAM-AS1 promotes colorectal cancer progression by acting as a molecular sponge of miR-384 to modulate AKT3 expression. *Aging (Albany NY)* 12:9781. 10.18632/aging.103243 32453706 PMC7288937

[B13] Liang J. Zhang S. Wang W. Xu Y. Kawuli A. Lu J. (2020). Long non-coding RNA DSCAM-AS1 contributes to the tumorigenesis of cervical cancer by targeting miR-877-5p/ATXN7L3 axis. *Biosci. Rep.* 40:BSR20192061. 10.1042/BSR20192061 31737900 PMC6944662

[B14] Liang W. H. Li N. Yuan Z. Q. Qian X. L. Wang Z. H. (2019). DSCAM-AS1 promotes tumor growth of breast cancer by reducing miR-204-5p and up-regulating RRM2. *Mol. Carcinog.* 58 461–473. 10.1002/mc.22941 30457164

[B15] Liao J. Xie N. (2019). Long noncoding RNA DSCAM-AS1 functions as an oncogene in non-small cell lung cancer by targeting BCL11A. *Eur. Rev. Med. Pharmacol. Sci.* 23 1087–1092. 30779076 10.26355/eurrev_201902_16998

[B16] Liu D. Rudland P. S. Sibson D. R. Barraclough R. (2002). Identification of mRNAs differentially-expressed between benign and malignant breast tumour cells. *Br. J. Cancer* 87 423–431. 10.1038/sj.bjc.6600456 12177779 PMC2376136

[B17] Liu F. Jia J. Sun L. Yu Q. Duan H. Jiao D. (2019). lncRNA DSCAM-AS1 downregulates miR-216b to promote the migration and invasion of colorectal adenocarcinoma cells. *OncoTargets Ther.* 12:6789. 10.2147/OTT.S213301 31686837 PMC6709378

[B18] Lu C. Xie T. Guo X. Wu D. Li S. Li X. (2020). LncRNA DSCAM-AS1 promotes colon cancer cells proliferation and migration via regulating the miR-204/SOX4 Axis. *Cancer Manag. Res.* 12:4347. 10.2147/CMAR.S250670 32606930 PMC7293419

[B19] Ma Y. Bu D. Long J. Chai W. Dong J. (2019). LncRNA DSCAM-AS1 acts as a sponge of miR-137 to enhance Tamoxifen resistance in breast cancer. *J. Cell. Physiol.* 234 2880–2894. 10.1002/jcp.27105 30203615

[B20] Miano V. Ferrero G. Reineri S. Caizzi L. Annaratone L. Ricci L. (2016). Luminal long non-coding RNAs regulated by estrogen receptor alpha in a ligand-independent manner show functional roles in breast cancer. *Oncotarget* 7:3201. 10.18632/oncotarget.6420 26621851 PMC4823100

[B21] Moradi M.-T. Fallahi H. Rahimi Z. (2020). The clinical significance of circulating DSCAM-AS1 in patients with ER-positive breast cancer and construction of its competitive endogenous RNA network. *Mol. Biol. Rep.* 47 7685–7697. 10.1007/s11033-020-05841-5 33040318

[B22] Niknafs Y. S. Han S. Ma T. Speers C. Zhang C. Wilder-Romans K. (2016). The lncRNA landscape of breast cancer reveals a role for DSCAM-AS1 in breast cancer progression. *Nat. Commun.* 7:12791. 10.1038/ncomms12791 27666543 PMC5052669

[B23] Ning Y. Bai Z. (2021). DSCAM-AS1 accelerates cell proliferation and migration in osteosarcoma through miR-186-5p/GPRC5A signaling. *Cancer Biomark.* 30 29–39. 10.3233/CBM-190703 32865178 PMC12499956

[B24] Park H. J. Ji P. Kim S. Xia Z. Rodriguez B. Li L. (2018). 3′ UTR shortening represses tumor-suppressor genes in trans by disrupting ceRNA crosstalk. *Nat. Genet.* 50 783–789. 10.1038/s41588-018-0118-8 29785014 PMC6689271

[B25] Qiu Z. Pan X. You D. (2020). LncRNA DSCAM-AS1 promotes non-small cell lung cancer progression via regulating miR-577/HMGB1 axis. *Neoplasma* 67 871–879. 10.4149/neo_2020_190826N821 32386483

[B26] Sun W. Li A. Q. Zhou P. Jiang Y. Z. Jin X. Liu Y. R. (2018). DSCAM-AS 1 regulates the G1/S cell cycle transition and is an independent prognostic factor of poor survival in luminal breast cancer patients treated with endocrine therapy. *Cancer Med.* 7 6137–6146. 10.1002/cam4.1603 30430768 PMC6308059

[B27] Tahmouresi F. Razmara E. Pakravan K. Mossahebi-Mohammadi M. Rouhollah F. Montazeri M. (2020). Upregulation of the long noncoding RNAs DSCAM-AS1 and MANCR is a potential diagnostic marker for breast carcinoma. *Biotechnol. Appl. Biochem.* 10.1002/bab.2048 [Epub ahead of print]. 33012018

[B28] Tarighi M. Khalaj-Kondori M. Hosseinzadeh A. Abtin M. (2021). Long non-coding RNA (lncRNA) DSCAM-AS1 is upregulated in breast cancer. *Breast Dis.* 40 63–68. 10.3233/BD-201010 33554879

[B29] Ulitsky I. Bartel D. P. (2013). lincRNAs: genomics, evolution, and mechanisms. *Cell* 154 26–46. 10.1016/j.cell.2013.06.020 23827673 PMC3924787

[B30] Wang N. Yang Y. Jia G.-Z. Wang K. Zhou S. Zhang B. (2021). Long non-coding RNA Down syndrome cell adhesion molecule-anti-sense 1 promotes gastric carcinoma cell proliferation and migration by regulating the miR-204/TPT1 axis. *Hum. Exp. Toxicol.* 09603271211036037. 10.1177/09603271211036037 [Epub ahead of print]. 34372727

[B31] Xu J. Wu G. Zhao Y. Han Y. Zhang S. Li C. (2020). Long noncoding RNA DSCAM-AS1 facilitates colorectal cancer cell proliferation and migration via miR-137/Notch1 Axis. *J. Cancer* 11 6623–6632. 10.7150/jca.46562 33046983 PMC7545673

[B32] Yin X. Wang P. Yang T. Li G. Teng X. Huang W. (2021). Identification of key modules and genes associated with breast cancer prognosis using WGCNA and ceRNA network analysis. *Aging (Albany NY)* 13:2519. 10.18632/aging.202285 33318294 PMC7880379

[B33] Yu C. Xu N. Jiang W. Zhang H. Ma Y. (2020). LncRNA DSCAM-AS1 promoted cell proliferation and invasion in osteosarcoma by sponging miR-101. *Eur. Rev. Med. Pharmacol. Sci.* 24 7709–7717. 32744697 10.26355/eurrev_202007_22274

[B34] Yue Z. Shusheng J. Hongtao S. Shu Z. Lan H. Qingyuan Z. (2020). Silencing DSCAM-AS1 suppresses the growth and invasion of ER-positive breast cancer cells by downregulating both DCTPP1 and QPRT. *Aging (Albany NY)* 12:14754. 10.18632/aging.103538 32716908 PMC7425442

[B35] Zhang Y. Huang Y.-X. Wang D.-L. Yang B. Yan H.-Y. Lin L.-H. (2020b). LncRNA DSCAM-AS1 interacts with YBX1 to promote cancer progression by forming a positive feedback loop that activates FOXA1 transcription network. *Theranostics* 10 10823–10837. 10.7150/thno.47830 32929382 PMC7482804

[B36] Zhang S. Ding L. Gao F. Fan H. (2020a). Long non-coding RNA DSCAM-AS1 upregulates USP47 expression through sponging miR-101-3p to accelerate osteosarcoma progression. *Biochem. Cell Biol.* 98 600–611. 10.1139/bcb-2020-0031 32379981

[B37] Zhang Y. Wang Y. Li C. Jiang T. (2020c). Systemic analysis of the prognosis-associated alternative polyadenylation events in breast cancer. *Front. Genet.* 11:1352. 10.3389/fgene.2020.590770 33329736 PMC7673440

